# Research on a Novel Fabry–Perot Interferometer Model Based on the Ultra-Small Gradient-Index Fiber Probe

**DOI:** 10.3390/s19071538

**Published:** 2019-03-29

**Authors:** Chi Wang, Jianmei Sun, Chenye Yang, Bin Kuang, Dong Fang, Anand Asundi

**Affiliations:** 1 Department of Precision Mechanical Engineering, Shanghai University, Shanghai 200444, China; wangchi@shu.edu.cn (C.W.); jianmeisun@shu.edu.cn (J.S.); yang_cheny@shu.edu.cn (C.Y.); BIN2016@shu.edu.cn (B.K.); 2 Science and Technology on Near-surface Detection Laboratory, Wuxi 214035, China; 3 School of Mechanical and Aerospace Engineering, Nanyang Technological University, Singapore 639798, Singapore; MASUNDI@ntu.edu.sg

**Keywords:** ultra-small GRIN fiber probe, F–P interferometer, optical fiber sensing technology, micro-vibration measurement

## Abstract

A novel Fabry–Perot (F–P) interferometer model based on the ultra-small gradient-index (GRIN) fiber probe is investigated. The signal arm of the F–P interferometer is organically combined with the ultra-small GRIN fiber probe to establish the theoretical model of the novel F–P interferometer. An interferometer experimental system for vibration measurements was built to measure the performance of the novel F–P interferometer system. The experimental results show that under the given conditions, the output voltage of the novel interferometer is 3.9 V at the working distance of 0.506 mm, which is significantly higher than the output voltage 0.48 V of the single-mode fiber (SMF) F–P interferometer at this position. In the range of 0.1–2 mm cavity length, the novel interferometer has a higher output voltage than an SMF F–P interferometer. Therefore, the novel F–P interferometer is available for further study of the precise measurement of micro vibrations and displacements in narrow spaces.

## 1. Introduction

With the development of precision interferometry, the miniaturization and integration of interferometers have become an important research direction. Fabry–Perot (F–P) fiber interferometers can achieve accurate measurements of physical parameters such as temperature [1,2], pressure [3], strain [4], and other physical parameters due to their small size, light weight, anti-electromagnetic interference, and high sensitivity. They are widely used in the health monitoring of large steel structures [5], deep oil well pressure measurements [6], and biomedical arterial blood pressure measurements [7], playing a major role in different fields such as industry, medicine, military, construction, aviation, and navigation [8]. However, most of the current F–P interferometers use a single-mode fiber (SMF) for signal arm transmission. For example, Zhou et al. studied a displacement sensor based on the F–P fiber interferometer [9], and Duan et al. studied an all-fiber F–P interferometer and its application in gas refractive index measurements [10]. Although this kind of signal arm can realize the miniaturization of the interferometer, the beam outputted by the SMF is divergent, causing problems such as a weak signal and a small dynamic test range during detection. In order to improve this situation, another study was conducted on the signal arm using an SMF connected to a self-focusing lens, such as the F–P interferometer system for micro-displacement measurements studied by Wei et al. [11]. However, its signal arm size is usually about a few millimeters, which is not suitable for the non-contact detection of sub-millimeter-sized components (such as micro-deep holes inside engines and gyroscopes) [12,13].

In view of this, a novel F–P interferometer model based on the ultra-small gradient-index (GRIN) fiber probe is studied. The GRIN fiber probe is an all-fiber optical probe consisting of a single-mode fiber, a coreless fiber, and a GRIN fiber [14,15]. Due to its ultra-small size and superior focusing performance, it can be integrated with the signal arm of the fiber interferometer through a fusion process, which has been favored by scholars in recent years [16,17,18,19]. In this paper, we study the ultra-small GRIN fiber probe samples with high coupling efficiency for the F–P fiber interferometer and their organic combination with the F–P fiber interferometer. Moreover, we build a new F–P interferometer detection experimental system and compare it with the single-mode fiber F–P interferometer. The results show that the novel F–P interferometer has a stronger output signal, a larger dynamic test range, and broad application prospects in high-precision measurements of micro-deep holes and other narrow spaces.

## 2. F–P Interferometer Model Based on the Ultra-Small GRIN Fiber Probe

**Figure 1:**
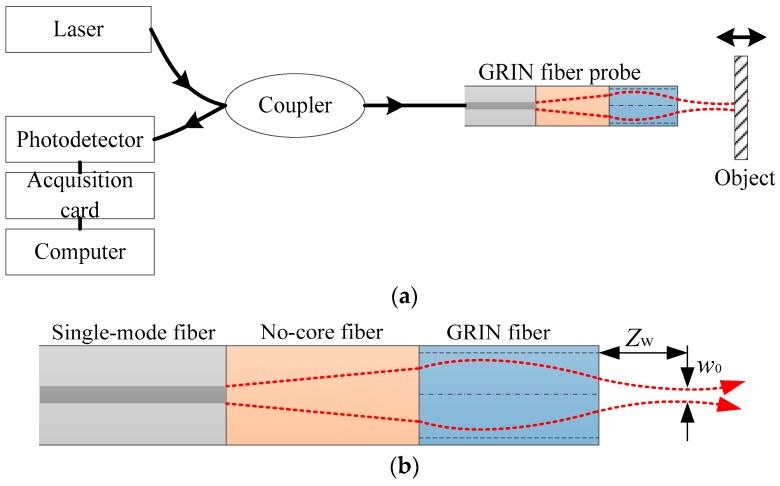
The schematic diagram of the Fabry–Perot (F–P) fiber interferometer based on the ultra-small gradient-index (GRIN) fiber probe: (**a**) the schematic diagram of the interferometer system; (**b**) the schematic diagram of the ultra-small GRIN fiber probe in the interferometer system.

The theoretical model of the novel F–P interferometer based on the ultra-small GRIN fiber probe is shown in Figure 1a. The model uses an ultra-small GRIN fiber probe as the interferometer detection arm, and the F–P interference cavity is formed between the probe output end face with an internal reflectance of 4% and the target reflection surface. The photodetector detects the signal and the signal is transmitted to a computer through a data acquisition card for analysis and processing. The ultra-small GRIN fiber probe model is shown in Figure 1b. *Z*_w_ represents the working distance (focal distance) of the GRIN fiber probe and *w*_0_ represents the focal spot diameter of the GRIN fiber probe. The length of the ultra-small probe is less than 1 mm and its diameter is 125 μm. If it is packaged with a fifth needle, its diameter is 0.5 mm. The probe consists of a single-mode fiber, a coreless fiber, and a GRIN fiber. Both the single mode fiber and the coreless fiber have a refractive index of 1.486. The GRIN fiber core has a core diameter of 50 μm, an outer diameter of 125 μm, a refractive index at the axis of 1.486, and a gradient constant of 5.587 mm^−1^. The coreless fiber expands the beam to overcome the problem of the small mode field diameter of the SMF, thereby improving the focusing performance of the GRIN fiber probe.

If the light beam is output from the GRIN fiber probe and incident vertically onto the reflective surface, the intensity of the signal that is finally output from the interferometer can be expressed as [20]:

(1) $$I_{R}=\frac{Q_{1}-Q_{2}\cos\phi }{Q_{3}-Q_{2}\cos\phi }I_{0}$$

where *Q*_1_ = *r*_1_^2^ + *r*_2_^2^, *Q*_2_ = 2 *r*_1_*r*_2_, *Q*_3_ = 1 + (*r*_1_*r*_2_)^2^, *r*_1_ and *r*_2_ are the reflection coefficients corresponding to the end face of the fiber and the reflection surface of the object to be tested, respectively, *ϕ* is the phase interferometer, and *I*_0_ is the incident light intensity. *I*_0_ is related to the output intensity of the light source, the insertion loss of the coupler, the loss of the transmission fiber, and the change of the polarization state. The phase difference *ϕ* includes an initial F–P cavity length caused by the initial phase difference *φ*_0_, a light source phase noise caused by the phase difference *φ*(*t*), a signal phase difference *φ*_s_(*t*), and the environmental noise caused by the phase difference *φ*_0_(*t*). It can be expressed as:

(2) $$\phi (t)=\varphi_{0}+\varphi (t)+\varphi_{s}(t)+\varphi_{0}(t)$$

If the object is under vibration, the distance between the object and the probe will change. The length of the F–P air cavity or the interference cavity will also change. Vibration information is modulated in the phase signal of the interfering light. The relationship between the cavity length change ∆*d* and the phase change of the interferometer ∆*_ϕ_* can be described as:

(3) $$\frac{\Delta_{d}}{\lambda }=\frac{\Delta_{\phi }}{4\pi }$$

where *λ* is the center wavelength of the source.

If the vibration signal of the object to be tested is a triangular wave vibration signal, the position of the triangular wave vibration signal at time *t* is shown as:

(4) $$\Delta l=\frac{8A}{\pi^{2}}\sum_{n=1}^{\infty }\frac{\sin(2n-1)\omega t}{{\left(2n-1\right)}^{2}}+A$$

where *A* represents the maximum amplitude of the vibration signal and *ω* represents the frequency of the vibration signal. By substituting Equation (4) into Equation (3), the formula can be described as:

(5) $$\Delta_{\phi }=\frac{32A}{\pi \lambda }\sum_{n=1}^{\infty }\frac{\sin(2n-1)\omega t}{{\left(2n-1\right)}^{2}}+\frac{4\pi A}{\lambda }.$$

The formula obtained by substituting Equation (5) into the phase *ϕ* in Equation (1) can be expressed as:

(6) $$I_{R}=\frac{Q_{1}-Q_{2}\cos\left(\frac{32A}{\pi \lambda }\sum_{n=1}^{\infty }\frac{\sin(2n-1)\omega t}{{\left(2n-1\right)}^{2}}+\frac{4\pi A}{\lambda }\right)}{Q_{3}-Q_{2}\cos\left(\frac{32A}{\pi \lambda }\sum_{n=1}^{\infty }\frac{\sin(2n-1)\omega t}{{\left(2n-1\right)}^{2}}+\frac{4\pi A}{\lambda }\right)}I_{0}.$$

**Figure 2:**
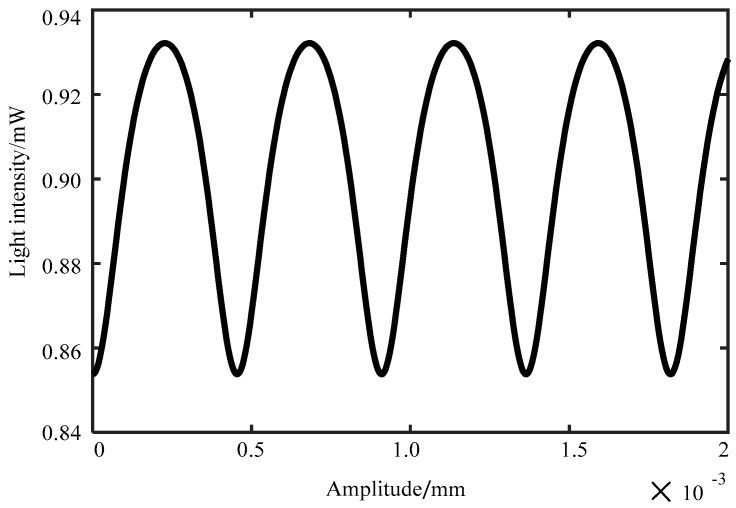
The relationship between interference intensity and amplitude.

**Figure 3:**
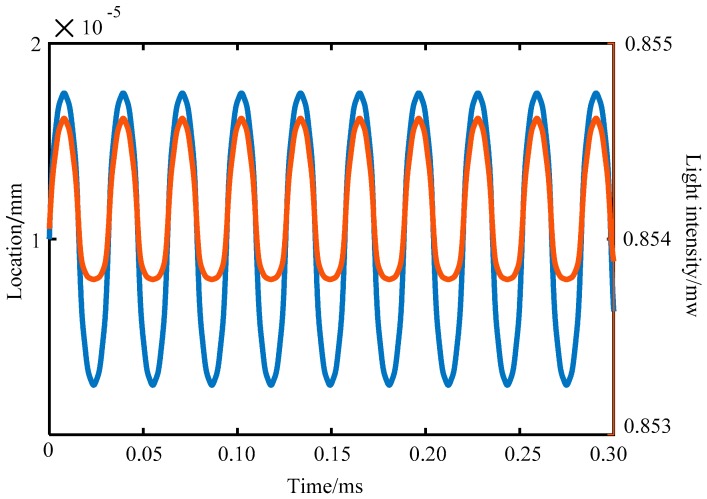
Vibration signal position and interferometer output light intensity variation with time.

According to Equation (6), when the reflection coefficient of the reflection surface, the wavelength of the incident light, and the light intensity are all determined, the interference intensity of the interferometer output is only related to the amplitude *A* and the frequency *ω* of the vibration signal at the same time. The vibration signal frequency *ω* is 200 Hz, the incident light intensity *I*_0_ is 1 mW, the wavelength *λ* is 1550 nm, *Q*_1_ is 0.94, *Q*_2_ is 0.076, *Q*_3_ is 1.036, and *n* is 5. At the same time, the output signal intensity of the interferometer varies with the amplitude of the vibration signal as shown in Figure 2. The signal intensity of the interferometer output varies with the amplitude of the vibration signal in an approximate cosine form. When the amplitude *A* of the vibration signal is fixed at 20 nm, the vibration signal (blue curve) and the output intensity of the interferometer (brown curve) change with time as shown in Figure 3. The vertical coordinate of the vibration signal curve is the vibration position, and its unit is in millimeters. The vertical coordinate of the output signal of the interferometer is light intensity, and its unit is in megawatts. As shown in Figure 3, the two curves have the same frequency. Therefore, the measurement of the amplitude and the frequency of the object to be measured can be converted into the measurement of the F–P interferometer’s output signal strength and frequency.

## 3. The F–P Fiber Interferometer Experimental System

**Figure 4:**
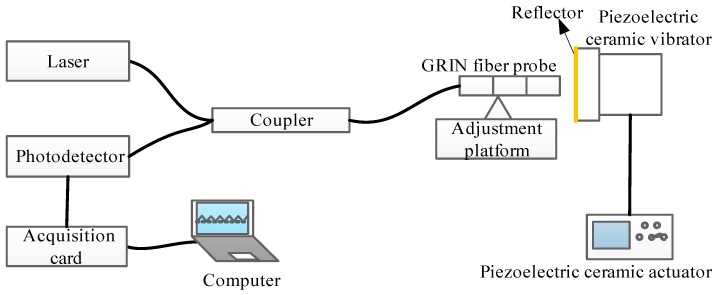
Schematic diagram of the experimental system of the F–P fiber interferometer.

**Figure 5:**
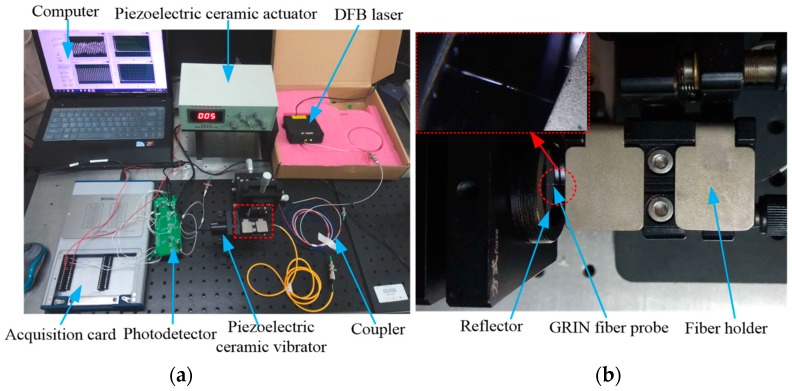
The F–P interferometer experimental system based on the ultra-small GRIN fiber optic probe: (**a**) picture of the experimental system; (**b**) a more detailed picture of the experimental system; (**b**) a larger view of the red dashed square from (**a**).

Based on the working principle of the F–P interferometer and the structural model of the miniaturized fiber interferometer, the experimental system of the F–P fiber interferometer shown in Figure 4 was constructed. Figure 5a is the corresponding picture of the experimental system. Figure 5b is a close-up view of the details circled in the red rectangle in Figure 5a. The system consists of a distributed feedback laser (DFB laser source), a 3 dB fiber coupler, a detection signal arm, a probe position adjustment platform, a piezoelectric ceramic vibration device, a photoelectric detection and signal processing unit, an acquisition card, and a computer. The main working principle is that the light emitted by the DFB source is split by the coupler and transmitted to the GRIN fiber probe, and is then incident perpendicularly to the mirror attached to the piezoelectric ceramic vibrator with a mirror through the GRIN fiber probe. Between the end surface of the fiber probe and mirror, after multiple reflections and transmissions, the reflected light is coupled into the GRIN fiber probe to generate interference. The interference signal with the vibration information is converted from the photodetector to an electric signal. The acquisition card collects the signal and transmits it to the computer.

In the experimental system of the interferometer shown in Figure 5, the GRIN fiber probe is the key component. Ordinary low-reflectivity external-cavity F–P fiber interferometers have some problems such as low reflectivity on the end surface and divergence of the light coming out from the SMF, so the distance between the two reflectors is very short, that is, the F–P cavity is small. Due to the limitation of the F–P cavity length, the dynamic measurement range of the external cavity interferometer is small. Therefore, according to References [15,18,19], we studied a kind of ultra-small GRIN fiber probe for the F–P interferometer. The coreless fiber length of the ultra-small GRIN fiber probe was 0.36 mm, the GRIN fiber length was 0.12 mm, the working distance was 0.506 mm, the focusing spot diameter was 30 μm, and the coupling efficiency at the working distance was 51%. The selected no-core fiber and GRIN fiber were both produced by Prime Optical Fiber Corporation. This probe can increase the dynamic measurement range based on the common F–P fiber interferometer, and its focusing performance is beneficial to increase the measurable signal bandwidth range.

## 4. Performance Detection of the Novel F–P Interferometer

**Figure 6:**
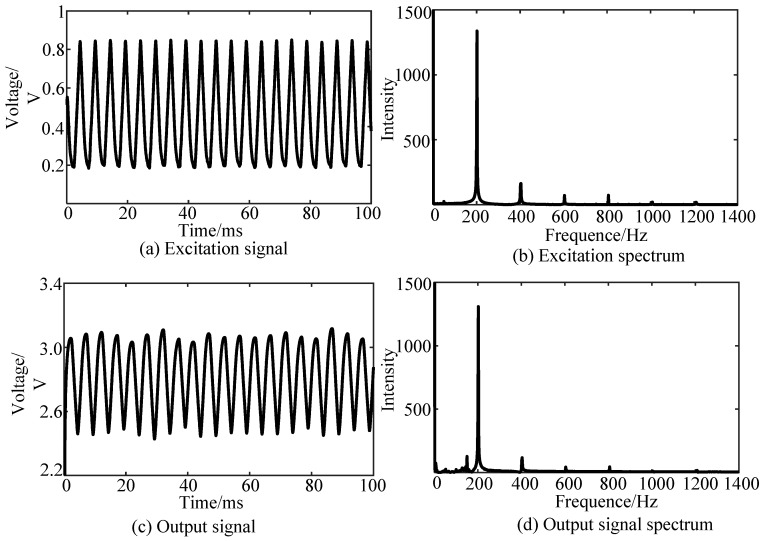
Time-domain and frequency-domain waveforms of the experimental signals at 200 Hz. They are: (**a**) excitation signal; (**b**) excitation spectrum; (**c**) output signal; (**d**) output signal spectrum.

According to the interferometer input–output relationship in Equation (6), the F–P interferometer experimental system shown in Figure 5 was applied to perform a micro-vibration measurement experiment. The driving frequency of the piezoelectric ceramic driver was set to 200 Hz. The excitation signal curve of the piezoelectric ceramic driver is shown in Figure 6a and can be used to compare and calibrate the measured signal. The interference waveform measured by the F–P interferometer is shown in Figure 6c. Figure 6b,d are spectrum diagrams of the excitation signal curve and the interference signal curve of piezoelectric ceramics, respectively. According to the spectrogram, the frequency of the measurement signal and the excitation signal are consistent and show good tracking characteristics.

It can be seen from Figure 6 that the F–P interferometer experimental system based on the ultra-small GRIN fiber probe has a good follow-up effect on the piezoelectric ceramic excitation signal. By comparing the frequency spectrum of the excitation signal with that of the interferometer measurement signal, it can be seen that the main frequency of the two signals is 200 Hz, and the intensity at their main frequencies is different. Therefore, the frequency of the low-frequency vibration signal can be detected by the F–P interferometer studied in this paper. In addition, compared with the excitation signal spectrum, the interferometer signal spectrum has some noise. According to the preliminary analysis, a slight change in the measurement environment may cause its vibration or change in position because the ultra-small GRIN fiber probe is not well packaged and is small in size and light in weight. In addition, the noise of the photodetector circuit may also be the cause of the signal frequency fluctuation.

**Figure 7:**
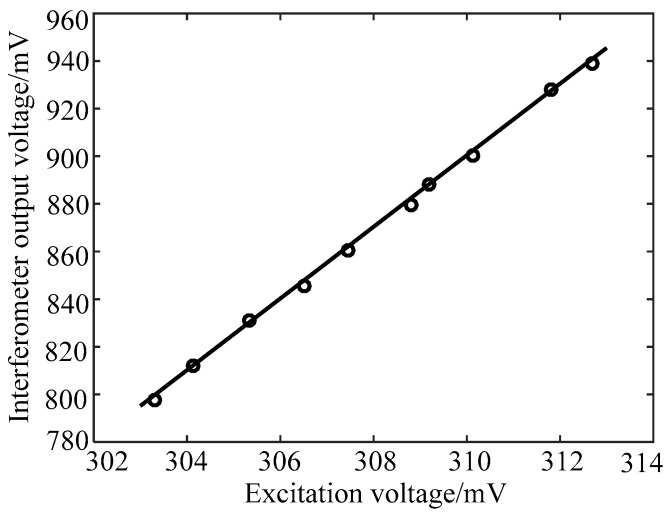
The relationship between the output voltage of the F–P interferometer and the excitation signal voltage.

It is known that the displacement and vibration amplitude of the piezoelectric ceramic vibrator are related to the excitation signal voltage of the piezoelectric ceramic driver. When the excitation signal frequency is fixed, the voltage variation of the excitation signal causes displacement or amplitude variation of the piezoelectric ceramic vibrator. The frequency of the excitation signal is 200 Hz. In the linear region of the interferometer, the output voltage of the F–P interferometer changes as the excitation signal voltage changes. Figure 7 shows the relationship between the output voltage of the F–P interferometer and the excitation signal voltage. The circled points represent the interferometer output voltage of the experimental acquisition, and the line is the curve of the variation relationship between two voltages. The fitting equation can be expressed as:

(7) y=x×15.04−3762

where *y* represents the interferometer output voltage and *x* represents the excitation signal voltage. The root mean square error of the fit between the two voltages is 1.306.

It can be seen from Figure 7 that the signal voltage of the F–P interferometer gradually increases with the increase of the excitation signal voltage. The relationship between them is linear. The linear sensitivity is that the voltage of the excitation signal increases by 1 mV, while the signal voltage of the F–P interferometer increases by 15.04 mV. Therefore, the F–P interferometer can perceive the amplitude of the vibration signal and the axial displacement of the piezoelectric ceramic vibrator. This characteristic can be applied to measure the small vibration and displacement of objects and provide a basis for the study of micro-vibration and displacement by using microfiber interferometers.

**Figure 8:**
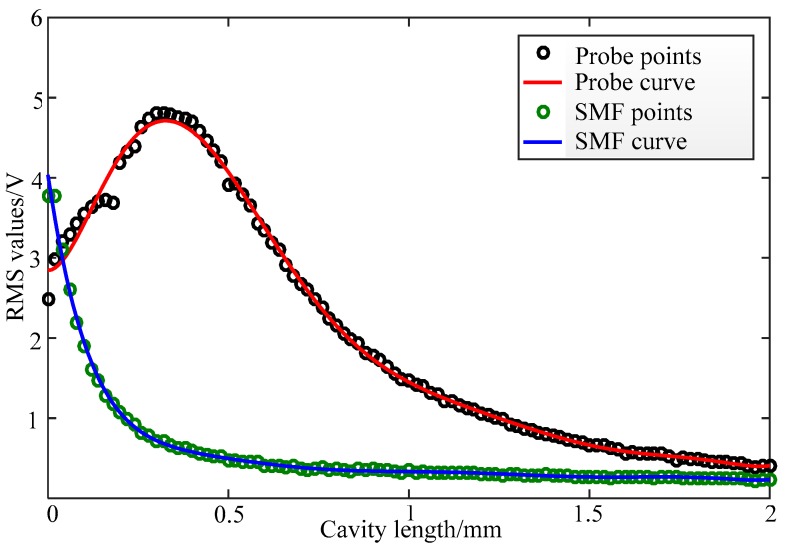
A comparison of the root mean square (RMS) values of the novel F–P interferometer and the ordinary F–P interferometer.

The novel F–P interferometer based on the ultra-small GRIN fiber probe and the ordinary F–P interferometer based on the single-mode fiber (SMF) were then experimentally compared. The output voltage of the interferometers was measured and the RMS (root mean square) value was calculated. Figure 8 shows the RMS value of two F–P interferometers varying in cavity length. The black circles are the RMS sample points of the novel F–P interferometer, and the red curve is the RMS curve obtained by fitting its sample points. The green circles are the RMS sample points of the ordinary F–P interferometer and the blue curve is the RMS curve obtained by fitting its sample points. It can be seen from Figure 8 that within the range of a 2 mm cavity length, the RMS value of the novel F–P interferometer first increases and then decreases with a maximum of 4.8 V. The RMS value is higher than 2 V in the range of a 0.8 mm cavity length. The root mean square value is 3.9 V at the working distance of 0.506 mm. For the ordinary F–P interferometer, the RMS value decreases monotonically and obtains its maximum of 4 V at the beginning. In the range of 0.1 mm cavity length, the RMS value is higher than the novel F–P interferometer. After that, the RMS values of the SMF F–P interferometer were relatively lower than those of the novel F–P interferometer. When the cavity length was increased to 0.2 mm, the RMS value decreased to 1 V. Therefore, the F–P cavity of the novel interferometer can be set to 0.5 mm, while the length of the F–P cavity of the ordinary SMF interferometer is about 0.1 mm, less than 0.5 mm. This further proves that the F–P interferometer based on the ultra-small GRIN fiber probe has superior focusing performance and a large dynamic measurement range.

## 5. Conclusions

In this paper, a novel F–P interferometer model based on an ultra-small GRIN fiber probe is studied and its experimental system is built. The output voltage RMS comparison with the SMF F–P interferometer is carried out. The results show that under the given conditions, the output voltage of the interferometer is linear with the excitation signal voltage and the sensitivity is 15.04. The output voltage is 3.9 V at a working distance of 0.506 mm, which is significantly higher than the 0.48 V of the single-mode fiber F–P interferometer at the same distance. Moreover, the output voltage of the new interferometer is higher than the output voltage of the SMF F–P interferometer in the range of 0.1–2 mm cavity length. It is demonstrated that the novel F–P interferometer based on the ultra-small GRIN fiber probe is feasible and has the advantages of a larger dynamic measurement range, a wider range of measurable signal bandwidth, and miniaturization. It can be used as a reference for the measurement of vibration amplitude and displacement with a novel F–P interferometer, and it comprehensively utilizes the characteristics of the ordinary F–P fiber interferometer and the superior focusing performance of the ultra-small GRIN fiber probe. The novel F–P interferometer has broader application prospects in micro-vibration measurements, micro-displacement measurements, and micro-deep holes endoscopic detection.
